# Advances in genome-wide RNAi cellular screens: a case study using the *Drosophila* JAK/STAT pathway

**DOI:** 10.1186/1471-2164-13-506

**Published:** 2012-09-24

**Authors:** Katherine H Fisher, Victoria M Wright, Amy Taylor, Martin P Zeidler, Stephen Brown

**Affiliations:** 1 The Sheffield RNAi Screening Facility, Department of Biomedical Science, University of Sheffield, Alfred Denny Building, Western Bank, Sheffield S10 2TN, UK; 2 The MRC Centre for Developmental and Biomedical Genetics and The Department of Biomedical Science, University of Sheffield, Firth Court, Western Bank, Sheffield S10 2TN, UK

**Keywords:** Genome screening, RNAi, Off-target effect, JAK/STAT pathway, Functional genomics, dsRNA

## Abstract

**Background:**

Genome-scale RNA-interference (RNAi) screens are becoming ever more common gene discovery tools. However, whilst every screen identifies interacting genes, less attention has been given to how factors such as library design and post-screening bioinformatics may be effecting the data generated.

**Results:**

Here we present a new genome-wide RNAi screen of the *Drosophila* JAK/STAT signalling pathway undertaken in the Sheffield RNAi Screening Facility (SRSF). This screen was carried out using a second-generation, computationally optimised dsRNA library and analysed using current methods and bioinformatic tools. To examine advances in RNAi screening technology, we compare this screen to a biologically very similar screen undertaken in 2005 with a first-generation library. Both screens used the same cell line, reporters and experimental design, with the SRSF screen identifying 42 putative regulators of JAK/STAT signalling, 22 of which verified in a secondary screen and 16 verified with an independent probe design. Following reanalysis of the original screen data, comparisons of the two gene lists allows us to make estimates of false discovery rates in the SRSF data and to conduct an assessment of off-target effects (OTEs) associated with both libraries. We discuss the differences and similarities between the resulting data sets and examine the relative improvements in gene discovery protocols.

**Conclusions:**

Our work represents one of the first direct comparisons between first- and second-generation libraries and shows that modern library designs together with methodological advances have had a significant influence on genome-scale RNAi screens.

## Background

The identification of RNAi and its implementation in cell culture has made systematic approaches to reverse-genetic screens a possibility [1]. Genome-wide RNAi screens in *Drosophila* cell lines, have identified genes involved in key cellular signalling pathways, such as Notch, JAK/STAT and Ras/MAPK [2-5]. However, major challenges are still associated with this kind of large-scale screening approach. Firstly, the failure to identify regulators (false negatives), due to reagent inefficiency, gaps in library design or cell type specific effects. Generally, false negative effects are unlikely to confound data processing. However, failure to identify genes that regulate the process of interest ultimately represents ‘lost’ information, which is thus not available for future analysis. The frequency of such false negatives can be reduced by improved reagent design and using multiple, independent RNAi reagents per gene [6,7]. The second challenge is the mistaken identification of genes - false positives that incorrectly appear to interact due to edge effects, liquid handling errors or the non-specificity of reagents (known as off-target effects (OTEs); [8,9]). Such false positives can make up more than 50% of primary screen data [6], are likely to confound initial analysis and can only be fully eliminated by downstream secondary screening and gene analysis *in vivo*.

One way to reduce the rate of false positives is via improvements in library design. Significant progress has been made in dsRNA design since the development of first-generation libraries such as the Heidelberg Fly Array (HFA) and *Drosophila* RNAi Screening Center (DRSC) v1.0 resources [10]. Current second-generation libraries, such as the Heidelberg HD2 library, generated by the Boutros (Heidelberg) and Kiger (UCSD) labs, have been designed to avoid OTEs predicted at the 19-nucleotide (nt) level. Indeed, a recent study reported that 37.1% of the first-generation HFA library dsRNAs contain potential OTEs, compared to 26.6% of HD2 library reagents [11]. Modern libraries also avoid repetitive elements, such as tandem repeats of the trinucleotide CAN (where N indicates any base) [8,12] with the frequency of dsRNAs that include CAN repeats being reduced from 5.3% in the HFA library to 0.5% in the second-generation HD2 library [11].

In addition to *in silico* design considerations, a number of additional advances have also been incorporated into the second-generation HD2 library. These involve a novel system of primer adaptors designed to minimise the chances of inter-well contamination and the use of dsRNAs targeting *DIAP1/thread* which are included in a pattern of wells, known as a ‘bar code’, unique to each library plate. *DIAP1* knockdown results in a strong cell death phenotype [13] and as a consequence, the pattern of ‘dead’ wells allows the post-screen identification of each library plate on the basis of cell survival (Additional file 1A) as well as serving as an indicator of dsRNA uptake and efficacy.

Although the use of second-generation libraries such as HD2, or the equivalent DRSCv2.0 [14], should give improved data quality, no published experimental analysis has been carried out to quantify these improvements using biologically comparable screens.

One of the few signalling pathways where multiple genome-wide RNAi screens have been completed, is the *Drosophila* JAK/STAT signalling pathway, where two first-generation library screens have been published [15,16] as well as a more recent screen using a customised commercial library [4]. These screens used different luciferase-based transcriptional reporters, cell lines and pathway stimulation protocols as well as significantly different bioinformatic post-screen processing (reviewed in [17]). Although all screens identified a number of core pathway components, the overlap of hits from the two first-generation screens was surprisingly small. However, the significant differences between the experimental approaches used prevent any systematic identification of factors responsible for the differences in gene lists ultimately identified. Indeed, low levels of overlap have also been reported for NF-κB signalling, which has also been repeatedly interrogated by RNAi screens, likely due to differences in reporters and cell types used [18].

For direct comparison of first- and second-generation libraries to be possible, identical screens using each library in parallel are required. However, due to the replacement, and hence the unavailability, of first-generation libraries this is no longer possible. Nonetheless, valuable comparisons can be made by comparing a substantively ‘similar’ screen to the data produced from a previous first-generation screen. Here we describe data derived from a new genome-wide RNAi screen for regulators of Upd-activated JAK/STAT signalling. This screen was undertaken using the HD2 second-generation dsRNA library as transcribed and reformatted in the Sheffield RNAi Screening Facility (SRSF). This screen is biologically as similar as possible to a previous screen undertaken using the first-generation HFA library [16]. We have analysed our new dataset using a defined set of rules employed by the SRSF as a standard, reproducible approach to screen analysis. These rules take advantage of the CellHTS2 R/Bioconductor package [19]. We have also used these rules to retrospectively reanalyse the original HFA screen-derived primary data, in order to eliminate differences in data processing from our comparison. We compare the results of the HFA- and HD2-derived screens and use these to both identify the genes involved in regulating JAK/STAT signalling and also to allow a comparison to be drawn between the first- and second-generation library screens.

## Results and discussion

### Genome-wide RNAi screening

**Figure 1:**
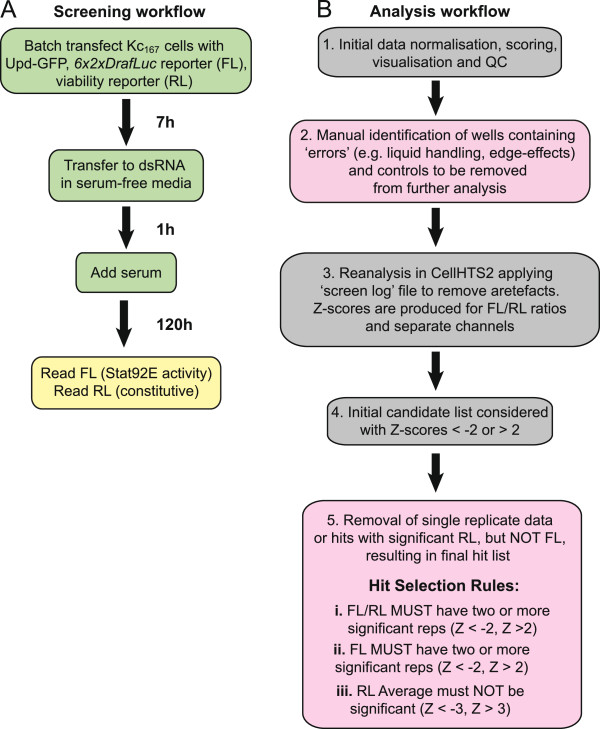
**Workflows of JAK/STAT RNAi screening methods and data analysis. (A) **Workflow of setting up the RNAi screen. Green boxes indicate steps carried out under sterile culture conditions. **(B)** Workflow of data analysis of genome RNAi data. Pink boxes indicate steps requiring manual data assessment and visualisation while grey boxes represent automated steps.

Ideally, a direct comparison of RNAi library designs would utilise two screens undertaken at the same time and in parallel that differ only in the libraries used. However, since the re-synthesis of a first-generation library to undertake such a direct comparison is not practicable, we set out to replicate a well-defined and previously published screen for which raw data was available. We therefore undertook a JAK/STAT RNAi screen modelled on a previous report carried out in 2005 using the first-generation HFA library (summarised in Figure 1A. See also Methods and [16] for details).

The principal differences between the original HFA and the repeated SRSF screen relate to the libraries used, and whilst the most obvious difference is to the sequences of the dsRNAs that make up the library itself, other factors may also be significant. For example, the plate layouts of the original HFA library (Additional file 1B) included 4 spaces per plate, which were used for controls targeting three positive pathway regulators (*dome, hop, Stat92E*) and the negative regulator (*Socs36E*). By contrast, the HD2 library was reformatted to allow additional duplicated controls as part of the library amplification undertaken at the SRSF (Additional file 1A) - changes that support independent statistical estimates of the required number of controls per plate [20]. This reformatted library is henceforth referred to as SRSFv1. Secondly, in order to add robustness and statistical confidence to the data produced, the new screen was repeated in triplicate, in contrast to the HFA screen, which was carried out in duplicate. For both HFA and SRSF screens, replicates were considered to be biologically independent of one another with a new batch of transfected cells used for each copy of the genome.

Given the differences in the libraries used, efforts were made to reproduce the biology of the original screen as closely as possible. Firstly, *Drosophila* Kc_167_ cells were batch transfected with the same quantities of a STAT92E-dependent transcriptional reporter (*6x2xDRafLuc*), pathway ligand to stimulate JAK/STAT pathway signalling (*pAc-Upd-GFP*) and a constitutive *Renilla* Luciferase reporter (*pAc-RLuc*), used to assess cell viability. Although the Kc_167_ cells used are derived from the same original source, precise matching of age and passage number between both screens could not be controlled. However, experience has shown that Kc_167_ cells are biologically stable with no detectable differences observed in their response to JAK/STAT signalling over at least 15 passages (data not shown). Following transfection, cells were transferred into library plates using automated liquid dispensers, and knockdown was allowed to occur over 5 days. Following cell lysis, luminometric substrates were added to measure both the Firefly Luciferase (FL) and Renilla Luciferase (RL) channels using a plate reader (Figure 1A).

### Data analysis

Many statistical methods are available for hit identification from large-scale screen data [21-23]. To be able to make comparisons between our SRSFv1-derived genome-wide screen and the original HFA screen, we analysed both raw datasets using the current ‘best practice’ SRSF analysis workflow (Figure 1B). All raw data was initially processed using the standard protocol for dual-luciferase based screens in the CellHTS2 package of R/Bioconductor [19]. This allows an initial assessment of overall data quality to be made by visualising the unfiltered data. Luciferase intensity ratios (FL/RL) were normalised by intra-plate median centering, then scored for significance using the robust Z-score (*Z* = *x*-median/MAD). In this approach the median absolute deviation (MAD) represents a measure of variation within the dataset that is less sensitive to outliers than other measures, such as standard deviation.

**Figure 2:**
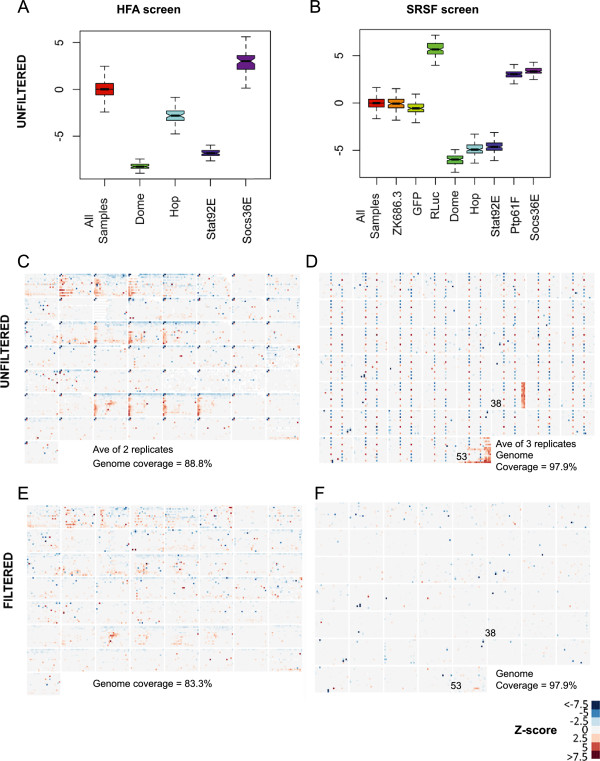
**Visualisation of whole genome data is required for error identification. (A-B) **Box and whisker plots show the averages and variance both of the whole data set (All Samples) as well as the controls used in the HFA screen and SRSF screens. GFP RNAi resulted in only a weak decrease in signalling, due to the high levels of Upd-GFP produced in the transfection. Failure of notches to overlap suggests that the two medians are significantly different. Y-axis shows Z-scores. **(C-F) **Heat maps representing Z-scores of FL/RL normalised values for the HFA screen (**C **and **E**) and the SRSF screen (**D **and **F**), in unfiltered format (**C **and **D**) and after filtering of controls and errors (**E **and **F**). As shown in the key, blues represent a decrease in pathway activity while reds indicate an increase.

In the first instance, controls included on each screening plate were assessed to determine the biological effectiveness of both screens (Figure 2A and 2B). In both screens positive controls known to be required for JAK/STAT signalling are recovered. Furthermore, the additional space available for controls in the SRSFv1 library also allows for technical controls (RLuc, which targets *Renilla luciferase* mRNA and thus skews the FL/RL ratio, and GFP, which targets the *Upd-GFP* mRNA) and the negative control (*C.elegans* dsRNA ZK686.3) to be added (Figure 2B). In addition, the effect of inter-plate and inter-replicate effects were also assessed using box and whisker graphs plotted for each plate (Additional file 2). When compared in this manner, the SRSFv1 data appears to be highly consistent - with the exception of plates 38 and 53 in replicate 3 (Additional file 2E, asterisks). By contrast, the HFA data shows considerably more variation between plates, a characteristic that often spans both replicates (Additional file 2A and 2B, asterisks).

Having confirmed that the controls had worked at a technical level, the Z-scores of each experimental replicate were then averaged and visualised as heat maps to identify technical errors whose inclusion would otherwise lead to false positives. For example, liquid handling errors on plates 38 and 53 of the SRSF screen (Figure 2D) and edge effects in rows A and B of several plates in the HFA screen (Figure 2C) are readily identified by human eye. In cases where liquid handling errors are identified, individual replicate data is examined to discover the source of the error. These evaluations, together with the known positions of control wells, are added to a ‘screen log’ file, a feature of the CellHTS2 package that allows wells to be excluded from downstream analysis. Following removal of the wells listed in the screen log file, Z-scores for the remaining wells are recalculated and plotted as heat maps which now show only interacting dsRNAs (Figure 2E and 2F). Significance was considered in wells with average FL/RL Z-scores greater than 2.0 or less than -2.0 (representing an equivalent p-value of <0.05; Figure 1B). However, it should be noted that while the triplicate data of the SRSF screen means that errors present in a single offending replicate could be removed (Figure 2F), the removal of edge effects in the HFA data leaves only one (or sometimes zero) remaining data point(s) (Figure 2E). Although not optimal, the number of wells excluded from the HFA dataset for this reason are included in Table 1 to facilitate inter-screen comparisons.

**Table 1:** HFA and SRSF genome coverage and hit selection

	**HFA screen**		**SRSF screen**	
	**FlyBase r2.0**		**FlyBase r5.24**	
	**Nr Genes**	**% of genome**	**Nr Genes**	**% of genome**
Initial Library coverage	13,226	88.8%	14,587	97.9%
Genes excluded due to screening errors (Figure 1B, step 2)	820	6.2%	0	0.0%
Remaining genome coverage	12,406	83.3%	14,587	97.9%
Genes with significant Average FL/RL (Figure 1B, step 4)	1,161	7.8%	300	2.0%
Final hits after applying all hit selection rules (Figure 1B, step 6)	134	0.9%	42	0.3%

The number of genes and % of the r 5.24 genome (14,898 genes) this represents are shown for each stage of the analysis pipeline. Genome annotation release used in the design of each library is indicated.

**Figure 3:**
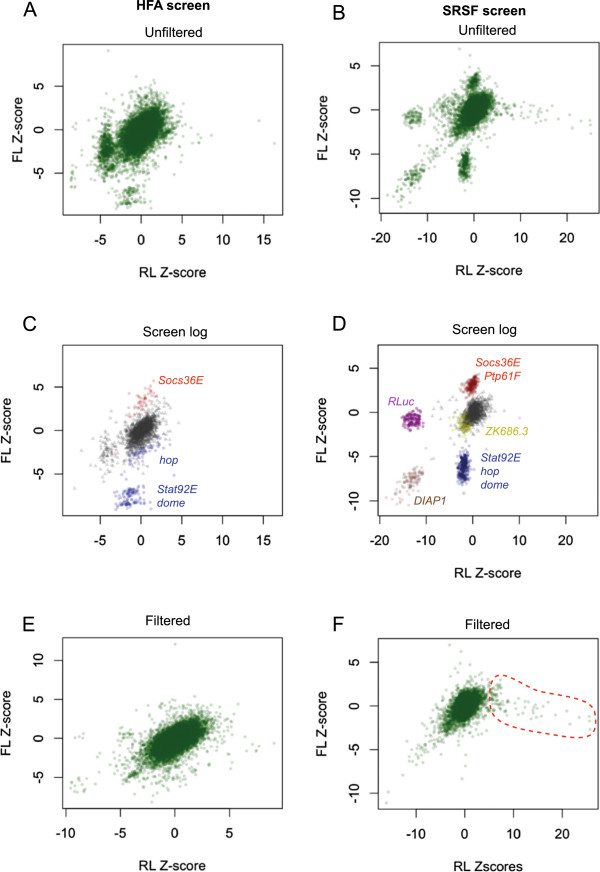
**Analysis of separate luciferase channels Scatter plots showing Z-scores of RL vs FL channels for HFA screen (A, C, E) and SRSF screen (B,D,F). **Wells from the screen log files, used to exclude data from the analysis, are shown for the HFA (**C**) and SRSF (**D**) screens. The positions of wells containing the indicated control dsRNAs are indicated in their corresponding colours, while grey triangles signify wells with errors or empty of dsRNA. (**E **and **F**) Show fully filtered data for each screen.

Analysis to this point has only considered the FL/RL ratios of potential interactions. This is a consequence of the screen design that uses a reporter constitutively expressing *Renilla luciferase* (RL) as a proxy for cell number. The prediction being that in a situation where JAK/STAT pathway activity is unaffected but cells under proliferate, both FL and RL channels are proportionately reduced with the FL/RL ratio remaining constant. However, this ‘biological normalisation’ relies on *luciferase* expression, activity and detection of both channels changing linearly with respect to one another. In cases where linearity is not perfect or values in either channel are extreme, the resulting FL/RL Z-score ratio can indicate a potentially misleading significant change (see Additional file 3). To visualise this relationship between the FL and RL signals, we plotted FL and RL channels separately for each screen (Figure 3), an approach that clearly visualises the effect of the controls used on both FL and RL channels (Figure 3C and 3D). Generally acting as anticipated, the pathway regulators (*hop, dome, Stat92E, Socs36E* and *Ptp61F*) effect the FL channel, but not on the RL channel (blue and red in Figure 3D), while the technical control targeting the *Renilla luciferase* mRNA has the inverse effect reducing RL levels but having no effect on FL (purple in Figure 3D). By contrast, the *DIAP1* control effects both channels (brown in Figure 3D), while the non-interacting control *ZK686.3* lies within the main cluster of samples (yellow in Figure 3D). We also visualise wells that were excluded due to edge effects or liquid handling errors during the initial data visualisation (triangles in Figure 3C and 3D). While many of these excluded wells cluster within the middle of the graphs (0,0), some are clearly outliers that may have been selected as putative hits without this level of analysis.

Although the biological and technical controls act as anticipated, we noticed that knockdown of a subset of genes initially characterised as hits actually show large RL Z-scores, but largely unaffected FL values (outlined in red in Figure 3F). Furthermore, visual monitoring of these wells in subsequent re-screens did not suggest that this increase in RL activity was a consequence of increased cell number (data not shown). Closer examination of the predicted biological ontologies of these genes identified multiple heat-shock proteins and components of the proteolysis pathway (Additional file 3). As such, it is likely that this interaction is actually a result of changes to *Renilla* Luciferase protein stability and/or activity. As such, additional steps to identify false positive signals arising from large (and potentially artefactual) changes in the RL signal are also necessary.

As a result of this finding, we included an additional hit selection criterion based on single channel data as a tool to identifying cell density artefacts misidentified as hits (Figure 1B). This additional selection rule requires wells with a significant FL/RL Z-score ratio in at least two replicates to also have significant Z-scores for the FL channel alone, while simultaneously not showing a significant Z-score in the RL channel (Figure 1B).

Following data analysis as described above and visually represented in Figure 1B, the SRSFv1 screen identified 42 putative hits representing 20 positive regulators and 22 negative regulators of pathway signalling (Table 2, Additional file 4). By contrast, reanalysis of the original HFA screen data using the same set of rules identified 134 putative regulators of pathway signalling (111 positive and 23 negative regulators; Additional file 5). The overlap between these groups and potential explanations for differences between them are examined below.

**Table 2:** Hits identified in SRSF RNAi screen and overlap with other screens

**Row**	**Gene**	**SRSF genome**	**SRSF secondary**	**HFA genome**	**Tertiary screen (independent dsRNAs)**
1	*dome*	**-7.9**	**-7.1**	**-8.0**	**-6.7**
2	*Stat92E*	**-6.8**	**-11.4**	**-6.5**	**-6.8**
3	*hop*	**-5.7**	**-10.0**	**-2.6**	**-4.4**
4	*mask*	**-4.3**	**-6.6**	**-3.8**	**NS**
5	*Mov34*	**-3.2**	**-4.1**	**-3.0**	0.1
6	*RpL24*	**-2.6**	**-2.5**	**-6.8**	0.1
7	*Ptp61F*	**2.3**	**3.6**	**5.0**	**4.1**
8	*CKIalpha*	**4.1**	**2.6**	**4.8**	**2.5**
9	*chinmo*	**6.6**	**4.8**	**7.0**	**2.7**
10	*TfIIA-L*	**-3.8**	**-5.5**	-1.8 #	**NS**
11	*Chd1*	**-2.2**	-1.7 #	**-2.4**	**NS**
12	*Saf-B*	**2.5**	1.8 #	**4.6**	**NS**
13	*Socs36E*	**2.8**	1.8 #	**2.5**	**3.9**
14	*shrb*	**2.3**	**3.0**	**NA**^**1**^	-0.4
15	*dom*	**-3.4**	**-2.4**	**NA**^**2**^	0.9
16	*RpLP2*	**-2.4**	**-2.2**	**NA**^**2**^	-0.5
17	*CG7185*	**2.6**	**1.7**	**NA**^**3**^	1.6
18	*RpII215*	**-2.2**	**-2.2**	**NA**^**3**^	-1.1
19	*Dp*	**-2.2**	**-2.8**	-2.8 *	-0.8
20	*CG32269*	**-3.1**	-1.7 #	-1.7 *	0.2
21	*E2f*	**-3.1**	**-4.9**	-1.0	-1.3
22	*CG11873*	**2.2**	**2.2**	0.0	**2.5**
23	*TSG101*	**2.3**	**6.0**	1.6	-1.1
24	*ftz-f1*	**2.3**	**2.7**	0.5	**2.0**
25	*kis*	**2.3**	**2.5**	0.9	0.0
26	*lola*	**3.1**	**4.0**	-0.8	-1.1
27	*srp*	**8.6**	**8.5**	-0.5	**NS**
28	*ctrip*	**-2.6**	-1.9 #	0.0	-1.1
29	*ham*	**4.5**	1.8 #	-0.5	1.7 #
30	*Tbp*	**-4.5**	0.5	-1.3	**-2.0**
31	*CG41021*	**-3.6**	-1.1	1.4	**-3.3**
32	*Dcp2*	**-2.0**	-0.6	-0.6	-0.4
33	*CG9723*	**-2.0**	1.3	-0.5	0.0
34	*pcnr017:3R*	**-2.0**	-0.4	1.2	-0.4
35	*Cnot4*	**2.1**	0.7	0.5	0.4
36	*qkr54B*	**2.2**	0.4	0.3	0.7
37	*l(3)mbt*	**2.6**	0.5	-0.4	0.8
38	*Hsp60B*	**2.7**	0.1	0.0	0.0
39	*Sin*	**2.7**	1.2	1.5	-0.1
40	*CG11399*	**2.8**	1.4	0.3	0.2
41	*Surf4*	**3.1**	0.8	1.3	-0.7
42	*zfh1*	**5.2**	0.8	0.6	0.6

**Bold** = Z-score <-2 or >2, # = Z-score <-1.7 or >1.7, * = only one replicate significant, NA = Excluded due to edge effects (1), error (2), error in one rep (3), NS = Not screened since previous clones are already independent designs.

### Screen comparison

Our analysis of the SRSFv1 screen and our reanalysis of the original HFA data set identified the known ‘core’ pathway components including the receptor *dome,* the JAK kinase *hop* and the *Stat92E* transcription factor as well as the negative regulator *Socs36E* and the tyrosine phosphatase *Ptp61F* (Table 2). However, despite the commonality at the level of the core pathway components, only 12 (29%) of the SRSF hits were also present in the reanalysed HFA data (Table 2, rows 1–13). The relatively small overlap between these apparently biologically similar experiments is unexpected given that the principal differences between the SRSF and HFA screens are the libraries used and the number of replicates screened. In addi-tion, we found that an even lower overlap, only 6 hits (Additional file 6), occurred between the SRSF and Baeg screens [15], a study undertaken using different reporter, cells and pathway activation methodologies. We therefore examined the HFA data relating to each of the 42 regulators identified in the SRSF screen to better understand why they were not selected as hits. Firstly, despite the reduced genome coverage of the HFA library, dsRNA designs targeting each of the 42 genes were present within the original HFA library. However, five of these genes were excluded, in one or both replicates, due to edge effects or liquid handling errors (marked NA in rows 14–18 of Table 2). Since our analysis rules require that at least two replicates must be significant, removal of one replicate in the HFA screen prevented the classification of these genes as a hit. A further two HFA targets gave significant, or close to significant, Z-scores (Table 2, rows 19–20) in only one replicate, a distribution that does not meet the cut-off criteria and so leads to these not being formally considered hits. As such, it is possible that the failure to identify these 7 genes is at least partly attributable to the lack of a triplicate dataset in the original HFA screen. The remaining 23 genes identified from the SRSF library, while present in the HFAs collection, were not found to be significant in the HFA screen (Table 2, rows 21–42).

To gain insight as to whether the 42 putative hits identified in the original SRSF screen represent false negatives from the HFA screen or false positives within the SRSF screen, we selected each of the 42 dsRNA PCR clones from the SRSF library, re-synthesised dsRNA and re-arrayed these with multiple non-interacting controls and rescreened using the same triplicate assay (Figure 1A). However, in this case, the large proportion of interacting wells present led us to modify data analysis by calculating Z-scores from the median of the non-interacting controls, rather than the whole plate (see Materials and Methods for details). Analysis of these repeated primary dsRNAs indicate that 22 of the original 42 hits are reproducibly significant in this assay (Bold in Table 2), with a further 7 trending in the same direction as the genome score with Z-scores <−1.7 or >1.7 (Hashes in Table 2).

Based on this secondary screen using the original dsRNA designs up to 29 genes represent potentially ‘true positives’ while 13 genes were not re-identified and so may represent false positives. This represents a potential false positive rate of 31%. Consistent with this classification, none of the putative false positive hits from the initial SRSF screen were found to be significant in the reanalysed HFA screen (Row 30–42 in Table 2). Considering only the validated hits identified in SRSF screens (Table 2 Rows 14–18) up to 68% of genes were also identified, or missed due to screening limitations, in the original HFA screen.

### Tertiary screening

While re-screening the original dsRNAs should eliminate technical variations, it does not provide an insight into the fidelity of the dsRNAs themselves. We therefore designed new dsRNAs targeting 37 of the 42 genes that were originally identified on the basis of a single dsRNA design (the remaining 5 having already been targeted by two independent dsRNAs present within the libraries are labelled NS [not screened] in Table 2). These new designs were generated by the E-RNAi tool [24] used to design the second-generation SRSF library but exclude the gene regions previously targetted (Additional file 5). Following dsRNA synthesis and quality control, this new set of reagents was used to undertake a tertiary re-screen using the same protocol already described. Following analysis, the core pathway components *dome*, *Stat92E* and *hop* were all re-identified as strong interactors as were the negative regulators S*ocs36E*, *Ptp61F*, *CkIalpha* and *chinmo* (Table 2). In total, 16 of the 42 hits (38%) were found to significantly interact with JAK/STAT signalling with two independent dsRNA reagents per gene. However, 15 of the new dsRNAs failed to interact significantly despite the original dsRNAs having been re-identified in both the original and secondary screen. The genes that were not re-identified include *TSG101*, a component of the endocytic trafficking machinery whose influence on JAK/STAT signalling has been previously examined in detail [25]. By contrast, the 15 genes that failed to interact also include two ribosomal proteins (*RpL24* and *RpLP2*) and the Polymerase II subunit *RpII215*, genes unlikely to represent JAK/STAT pathway-specific interactors.

Tertiary screening also provided insights into the 13 genes only identified in the primary genome-scale screen (rows 30–42 in Table 2). Two genes (*Tbp and CG40121*) were re-identified in the tertiary screen – suggesting that these are in fact legitimate regulators and false negatives in the secondary screen. While the weight of evidence suggests that the remaining 11 genes were primary false positives, it remains possible that genuine interactors remain. For example, *Cnot4* has previously been shown to act as a pathway regulator in S2 cells and has been validated using cell-based and *in vivo* assays [26]. Taken together, this tertiary screening highlights not only non-specific effects of the original library, but also the potential for reagent efficiency and experimental variability to confound results in this class of experiment.

To clarify whether similar inconsistencies occurred within the HFA library, we also re-screened independent dsRNA reagents targeting 126 of the 134 hits from the HFA genome-wide screen. We were unable to design, or had previously screened and found to be significant, independent reagents targeting the remaining 8 (Additional file 5). Fifty of the independent designs were picked as PCR templates from the SRSFv1 library, and the remaining 77 were newly designed. Of the 128 genes screened with two independent dsRNAs, only 8 showed an interaction consistent with the HFA genome-wide data, with a further 4 trending in the right direction (Additional file 5). This remarkably low overlap of only 6%, suggests that a large proportion of the HFA ‘hits’ were due to non-specific effects.

### Off-target analysis

Although RNAi reagents can behave with high specificity in *Drosophila* cells, as previously shown by microarray experiments [27], OTEs can represent a potential source of false positive hits in RNAi-based experiments. However, modern *in silico* library design approaches are beginning to reduce their impact and *in silico* off-target prediction approaches suggest that only 26.6% of the dsRNAs that make up the HD2 library have potential OTEs at the 19-nt level, compared to 37.1% in the first-generation HFA library [11]. We have used the data from the HFA and SRSF JAK/STAT screens, and the predicted OTEs for each of these hits, to investigate this further. Firstly, the 134 hits found in the HFA reanalysis and the 42 hits from the SRSF genome screens have a similar frequency of one or more predicted OTEs, 44.8% v. 44.1%, respectively. To define whether these potential OTEs could influence the identification of interacting genes, we generated a list of interacting dsRNAs, their primary intended targets and their potential off-targets as predicted at the 19-nt level by NEXT-RNAi [11]. We then investigated whether any of these predicted off-targets appear as primary hits in either of the two genome screens. Such a scenario would suggest that the interaction of the original dsRNA might be a consequence of the off-target and thus represent a false positive JAK/STAT pathway regulator. Of the 134 genes (136 dsRNA clones) identified from the HFA screen, we found that 10 have predicted OTEs that target a gene identified in either screen (7.4%) (Table 3). By contrast, of the 42 genes (43 dsRNA clones) in the SRSF hit list, only one dsRNA had predicted off-targets that were also hits in one of the screens (2.3%) (Table 3). This indicates that while a similar number of clones with potential OTEs are identified in the primary screens, those whose OTEs are likely to have effected the final outcome are less frequent in the SRSF-derived data.

**Table 3:** Off-target analysis of HFA and SRSF hit lists

**On target**			**Predicted off target**				
**On target - dsRNA**	**On target - Gene**	**On target - score**	**Off target – Flybase ID**	**Off target - Gene**	**Off target - dsRNA**	**Off target - score**	
HFA16984	*larp*	-3.1	**FBgn0003013**	***osa***	**HFA17022**	**-4.2**	*
HFA18710	*RpS14a*	-6.1	**FBgn0004404**	***RpS14b***	**HFA18711**	**-4.9**	*
HFA19901	*CG4411*	-2.3	FBgn0039633	*CG11873*	BKN26700	2.2	
			**FBgn0028991**	***CG4055***	**HFA06863**	**-3.7**	*
			**FBgn0043884**	***mask***	**HFA16018**	**-5.6**	*
			**FBgn0043884**	***mask***	**HFA16005**	**-5.8**	*
			**FBgn0043884**	***mask***	**BKN20625**	**-4.3**	*
			**FBgn0004656**	***fs(1)h***	**HFA18778**	**-4.1**	*
			FBgn0086902	*kis*	BKN20986	2.3	
			**FBgn0004864**	***hop***	**HFA20340**	**-5.8**	*
			**FBgn0004864**	***hop***	**BKN24272**	**-5.7**	*
HFA12365	*Rm62*	3.1	**FBgn0035720**	***CG10077***	**HFA09691**	**3.1**	*
HFA07091	*CG8179*	4.9	FBgn0020306	*dom*	BKN21379	-3.4	
			FBgn0015618	*Cdk8*	HFA11113	-2.3	
			**FBgn0015903**	***apt***	**HFA04671**	**3.2**	*
			FBgn0026575	*CG4411*	HFA19901	-2.1	
			FBgn0260794	*ctrip*	BKN20611	-2.6	
			FBgn0028991	*CG4055*	HFA06863	-3.7	
			**FBgn0039633**	***CG11873***	**BKN26700**	**2.2**	*
			FBgn0000008	*CG14169*	HFA10170	-2.2	
			FBgn0003013	*osa*	HFA17022	-4.2	
			**FBgn0051716**	***Cnot4***	**BKN22063**	**2.1**	*
			FBgn0260724	*larp*	HFA16984	-2.9	
			**FBgn0035720**	***CG10077***	**HFA09691**	**3.1**	*
			FBgn0011666	*msi*	HFA17003	-2.6	
			**FBgn0003507**	***srp***	**HFA17068**	**13.6**	*
			**FBgn0003507**	***srp***	**BKN45799**	**8.6**	*
			FBgn0004656	*fs(1)h*	HFA18778	-4.1	
			FBgn0003687	*Tbp*	BKN20836	-4.5	
			FBgn0012049	*msi*	HFA17003	-2.6	
			**FBgn0004606**	***zfh1***	**BKN29931**	**5.2**	*
			**FBgn0001078**	***ftz-f1***	**BKN28995**	**2.3**	*
			**FBgn0086902**	***kis***	**BKN20986**	**2.3**	*
			FBgn0034258	*CG4954*	HFA06905	-5.5	
			FBgn0027492	*CkIIbeta*	HFA20230	-2.2	
			FBgn0032633	*CG4055*	HFA06863	-3.7	
			FBgn0043884	*mask*	HFA16018	-5.6	
			FBgn0043884	*CG6268*	HFA16005	-5.8	
			FBgn0043884	*mask*	BKN20625	-4.3	
HFA06863	*CG4055*	-4.2	**FBgn0015618**	***Cdk8***	**HFA11113**	**-2.3**	*
			**FBgn0000008**	***CG14169***	**HFA10170**	**-2.2**	*
			FBgn0003507	*srp*	HFA17068	13.6	
			FBgn0003507	*srp*	BKN45799	8.6	
			FBgn0039633	*CG11873*	BKN26700	2.2	
			FBgn0001078	*ftz-f1*	BKN28995	2.3	
			FBgn0086902	*kis*	BKN20986	2.3	
HFA04671	*apt*	2.7	**FBgn0001078**	***ftz-f1***	**BKN28995**	**2.3**	*
			FBgn0030636	*cngl*	HFA19089	-2.7	
			**FBgn0003507**	***srp***	**HFA17068**	**13.6**	*
			**FBgn0003507**	***srp***	**BKN45799**	**8.6**	*
HFA18711	*RpS14b*	-6.0	**FBgn0004403**	***RpS14a***	**HFA18710**	**-4.9**	*
HFA17068	*srp*	8.5	FBgn0030636	*cngl*	HFA19089	-2.7	
			FBgn0020306	*dom*	BKN21379	-3.4	
			FBgn0015618	*Cdk8*	HFA11113	-2.3	
			**FBgn0039633**	***CG11873***	**BKN26700**	**2.2**	*
			FBgn0260794	*ctrip*	BKN20611	-2.6	
			**FBgn0003016**	***kis***	**BKN20986**	**2.3**	*
			**FBgn0086758**	***CG17156***	**HFA00485**	**7.0**	*
			**FBgn0086758**	***CG17649***	**HFA00509**	**7.0**	*
			**FBgn0086758**	***chinmo***	**BKN45751**	**6.6**	*
			**FBgn0005630**	***lola***	**BKN30256**	**3.1**	*
			FBgn0000259	*CkIIbeta*	HFA20230	-2.2	
			FBgn0028371	*CG13525*	HFA04167	-2.9	
			FBgn0032633	*CG4055*	HFA06863	-3.7	
			**FBgn0015903**	***apt***	**HFA04671**	**3.2**	*
			FBgn0026575	*CG4411*	HFA19901	-2.1	
			**FBgn0001078**	***ftz-f1***	**BKN28995**	**2.3**	*
			FBgn0011666	*msi*	HFA17003	-2.6	
			FBgn0028991	*CG4055*	HFA06863	-3.7	
			**FBgn0035720**	***CG10077***	**HFA09691**	**3.1**	*
			FBgn0000008	*CG14169*	HFA10170	-2.2	
			**FBgn0086902**	***kis***	**BKN20986**	**2.3**	*
HFA09691	*CG10077*	2.6	**FBgn0003261**	***Rm62***	**HFA12365**	**3.8**	*
BKN41059	*CG40121*	-3.6	**FBgn0043903**	***dome***	**BKN25660**	**-7.9**	*
			**FBgn0000259**	***CkIIbeta***	**HFA20230**	**-2.2**	*
			**FBgn0004656**	***fs(1)h***	**HFA18778**	**-4.8**	*
			**FBgn0043903**	***dome***	**HFA19583**	**-13.5**	*
			**FBgn0010412**	***RpS19***	**HFA20281**	**-7.0**	*

Bold and * = labelling of off-targets indicates that their knockdown resulted in a significant Z-score in the same direction as the original intended gene.

On-target dsRNAs with potentially interacting off-targets identified in each screen.

The frequency of interacting OTEs found in our screen is either a random effect or a consequence of selection imposed by the screen itself. To investigate this, we randomly selected 100 dsRNAs from each library and counted how many of the associated predicted off-targets appear within the same list of 100 primary gene targets. After 1000 such search iterations, we find that the SRSF library has significantly fewer ‘circular’ OTEs (0.5%) compared to the HFA library (3.4%), a result that demonstrates the improvements in the SRSF/HD2 library design. However, this random ‘background’ chance of circular OTEs is significantly lower than the actual rate of potential OTEs found in our gene lists and suggests that the biological selection imposed by a screen is a more important factor in determining the frequency of OTEs encountered in practice.

## Conclusions

Here we present the results of a screen undertaken at the Sheffield RNAi Screening Facility (SRSF), one of only a handful of *Drosophila* RNAi screening facilities open to external screeners [28]. In order to be able to qualitatively evaluate the performance of the second-generation SRSFv1 library used at the SRSF we set out to replicate a previous genome-wide RNAi screen designed to identify regulators of the JAK/STAT signalling cascade [16]. This original screen used a first-generation library of dsRNAs (Table 1) and identified interacting loci that have since been extensively investigated and validated [29], including the phosphatase Ptp61F and the positive regulator BRWD3 [16]. Given the provenance of the original gene list, we therefore expected the SRSF screen results to correlate closely, especially given that both data sets were analysed using equivalent bioinformatic rules within this study (Figure 1B). Indeed, the technical quality of the triplicate SRSF screen data appears to be very high (Additional file 2C-E), and represents a significant improvement over the original HFA data (Additional file 2A-B, also compare Figure 2C and 2D). Furthermore, the triplicate data set allows a degree of robustness in downstream rejection of edge effects and similar artefacts unavailable to the duplicated HFA data, an observation that provides confidence in the veracity of the SRSF results generated.

Surprisingly however, initial comparisons of the SRSF and HFA screen results suggested that as little as 29% (12 of 42) of the SRSF hits were common to both. Following secondary screening of the 42 genes initially identified by the SRSF genome-wide screen, only 22 genes were re-identified at high confidence levels, representing a 48% false positive rate for the SRSF screen. A further 7 genes were found to be trending in the same direction as in the genome-wide screen, whose consideration increases the potential hit list to 29 genes and decreases the false positive rate to 31%. Taking into account marginal strength interactions and genes with missing data it is possible that up to 20 of 29 interacting loci identified by the SRSF screen were also present in the original HFA dataset (Table 2), a rate of 68%. Furthermore, even excluding ‘potential’ overlaps where Z-scores fall just below the -2 cut off, or instances where not all HFA data is available, an overlap of 60% is still obtained (12 of 22). By contrast, the levels of overlap to the larger HFA gene list are smaller. In part this is expected, as secondary screening of these primary HFA hits is not possible given the unavailability of the original HFA library clones. In addition, it is also likely that the availability of only two data points, together with the higher levels of experimental noise in the HFA data (Additional file 2) conspire to increase the rate of false positives in this primary list.

The tertiary screen used new independent dsRNA designs targeting 37 of the 42 genes previously targeted with a single dsRNA (Table 2). Taken together with the 5 genes previously confirmed by multiple dsRNAs, 38% (16 of 42) of the putative interactors were confirmed by the tertiary analysis. As expected, this tertiary analysis also excluded most of the genes previously rejected as primary false positives by the secondary and HFA screens. However, tertiary analysis also failed to re-identify 45% (9 of 20) of the genes identified by primary, secondary and HFA screens as well as 48% (14 of 29) of those identified by only the primary and secondary screens. This relatively low rate of re-identification may result from either false positive in the original screen or false negatives in the tertiary. In particular, the design of the tertiary dsRNAs (Additional file 5), which are obliged to avoid the regions used by the SRSFv1 dsRNA designs, may be less efficient and so more likely to generate false negative results. Despite this, the designs used in the tertiary screening successfully identified the core JAK/STAT components, such as *dome*, *hop*, *Stat92E*, *Ptp61F* and *Socs36E* and excluded ribosomal proteins and core RNA polymerase II subunits unlikely to represent true positives. Tertiary screening also served to reconfirm *Tbp* and *CG40121* as *bona fide* interactors, genes that were not confirmed in the secondary screens - presumably due to false negative effects during the secondary screen. Remarkably, tertiary analysis of the HFA screen hits revealed that, apart from the core pathway components mentioned, only 8 genes (6%) were reconfirmed with independent dsRNA designs (Additional file 5). This suggests that the SRSF library does indeed represent a significant improvement over this first-generation library.

Although technical differences in experimental design undoubtedly play a part in creating these differing hit lists, a significant factor are recent advances in dsRNA library design. While the technical background to second-generation library design has been reported elsewhere [11], our work represents one of the first direct comparisons between first- and second-generation libraries. Of particular interest in this respect is the identification of ‘off-target’ effects that knock down an unintended secondary mRNA in addition to the primary ‘on’ target. In order to establish a baseline of theoretical OTEs we repeatedly generated and tested random lists of library dsRNAs and searched for internal OTEs within this group. This suggested that a hit list derived from the HFA library would include 3.4% potential off-target false positives while the SRSF library would only include 0.5% off-target clones. By contrast, our biological data shows significantly higher off-target rates than predicted with 7.4% (10 of 136; HFA) and 2.3% (1 of 43; SRSF) predicted off-target clones being identified (Table 3). We suggest that this experimentally observed enrichment is likely to be a consequence of screening itself, with the search for modulators of JAK/STAT signalling specifically enriching for genes with OTEs able to modulate pathway activity. As a consequence, a screen will automatically enrich for dsRNAs with interacting OTEs and an increased frequency of off-target clone identification is largely unavoidable and is likely to increase as the assay improves. This highlights the importance of using improved libraries optimised to minimise off-target effects and also demonstrates the utility of post-screen *in silico* analysis to identify false positive hits resulting from potential OTEs.

Finally, it should also be highlighted that despite the apparent reproducibility of the primary SRSF data over three independent biological replicates, 31% (13 of 42) of genes initially identified were subsequently classified as false positives following rescreening. However, two of these apparently false positives were subsequently re-identified by tertiary screening and so actually represent secondary false negatives. The added variability of dsRNA potency and efficiency, as well as differences between dsRNA preparations may all play a part in these inconsistencies. As such, the emphasis on secondary and tertiary screens, using multiple dsRNA designs, different assays and using information about the transcriptome of the cells being screened remains an essential aspect of any genome-scale experimental design.

Overall, we have established an improved framework for the design and implementation of RNAi screens using currently available libraries and analysis methods. In addition, the iterative process of screening and analysis has refined our understanding of genes regulating *Drosophila* JAK/STAT signalling*,* revealing novel players in the process. However, even the most sophisticated screening approaches are only a tool to identify genes that potentially interact with a chosen assay system. Ultimately, downstream validation, analysis and investigation are required to confirm the true functional and physical nature of the biological networks being studied.

## Methods

### SRSFv1 dsRNA library production

The SRSFv1 library was synthesised at the SRSF, from PCR products kindly provided by Michael Boutros from the HD2 collection. Details of the probes used in the HD2 and SRSF libraries can be found at http://www.dkfz.de/signaling/nextrnaiData/calc/HD2/out/. Synthesis was carried out according to [30]. Briefly, PCR products were amplified using T7 primers using Reddymix (Abgene) according to manufacturers instructions. PCR products were checked for size of single bands by running on E-gel Precast Agarose Gels (Invitrogen). Any PCRs that failed to give a product were repeated. dsRNA was produced using the T7 MEGAscript Kit (Ambion) according to manufacturer’s instructions and incubated for 16 h at 37°C. DNAse I treatment removed the PCR template and then an ethanol precipitation was carried out. RNA pellets were eluted in water, checked by running on a gel and quantified using a Nanodrop. dsRNA was then diluted in water 27-fold to a working concentration in the range 20–200 ng/μl. Of this dilution, 5 μl (100 ng – 1 μg) was added per well of a 384-well plate in each screen. Screening plates were sealed, with an Agilent PlateLoc plate sealer, and stored at-80°C until needed. The HD2 library was reformatted to allow for an increased number of controls per plate. For an example plate layout see Additional file 1A.

### Design of tertiary dsRNAs

Newly designed dsRNAs for tertiary screening were designed using the E-RNAi webservice v3.2 [24] and are described in Additional file 5. Original dsRNA regions were avoided using the options within the web tool.

### Cell culture and RNAi screening

*Drosophila* Kc_167_ cells were cultured under standard conditions at 25°C, in Schneider’s media (Gibco) supplemented with 10% FBS (Sigma) and 1% Penicillin/Streptomycin (Gibco).

For screening, *Drosophila* Kc_167_ cells were grown to almost confluence in T-75 flasks and were then passaged into 9xT-75 flasks at a density of 40 million cells per flask and allowed to recover overnight. Cells were then transfected with *6x2xDRafLuc*, *pAc-RLuc*, *pAc-Upd-GFP*, and *pAc* empty vector as originally described in [16] using Effectene (Qiagen) according to manufacturers instructions. The cells were then incubated for 7 h, after which media was replaced with fresh media lacking serum. Cells were then seeded into the 384-well library plates, using an automated liquid dispenser (Multidrop, ThermoFisher), at a density of 15,000 cells per well in 20 μl of serum-free media. After 1 h, 10 μl of media, supplemented with 30% serum, was added to each well and the plates sealed and incubated for 5 days at 25°C. The genome screen was replicated in completely separate biological triplicates with an interval of several weeks between each replicate.

### Luciferase assays

RNAi-mediated knockdown was allowed to occur over 5 days, after which luciferase activity was measured as previously described [31]. The plates were vibrated for 5 s prior to reading on a Varioskan (ThermoFisher) plate reader at 100 ms acquisition time per well. The 53 plates containing the genome were processed in batches of 17 or 18 plates and data files were produced per channel for each batch.

### Data analysis

Statistical analysis was completed using the following packages: R64 version 2.12.0, biocinstall version 2.7.7, Bioconductor version 2.7 and cellHTS2_2.14.0. R scripts were run on a Mac OS10.6 operating system. The data files were deconvolved into 318 individual plate .txt files for each firefly and renilla luciferase channel, using a Perl sub-routine, ready for analysis within the CellHTS2 package in R/Bioconductor. Perl and R scripts available on request.

HFA library annotation has been updated, with detailed information provided at http://www.dkfz.de/signaling/nextrnaiData/calc/HFA/out/. Data analysis was carried out as for the SRSF data.

## Abbreviations

RNAi: RNA-interference; Min: Minutes; H: Hours; dsRNA: Double stranded RNA; mRNA: Messenger RNA; SRSF: Sheffield RNAi Screening Facility; DRSC: *Drosophila* RNAi Screening Centre; OTE: Off-target effect.

## Competing interests

The authors declare that they have no competing interests.

## Authors’ contributions

KF performed data analysis and interpretation and helped to draft the manuscript. VW designed and optimised the screening assay. AT performed SRSF screens. MZ conceived of the study, assisted in its design and coordination and helped to draft the manuscript. SB performed SRSF screens, assisted in the design and coordination of the study, performed data analysis and interpretation, and helped to draft the manuscript. All authors read and approved the final manuscript.

## Supplementary Material

Additional file 1 **Plate layouts of HFA and SRSFv1 libraries. **(A) Layout of HFA library plates including the position of positive (blue) and negative pathway regulators, used as controls in the Müller et al. screen. (B) Layout of SRSFv1 library including the *DIAP1 *barcode (black), technical controls (purple), non-interacting controls (yellow) and positive (blue) and negative (red) pathway regulators used as controls. Click here for file

Additional file 2 **Quality control of HFA and SRSF JAK/STAT screens. **Box and whisker plots representing each plate from separate replicates in HFA and SRSF screens. Asterisks denote plates where variance can be observed by eye. Click here for file

Additional file 3 **dsRNAs resulting in high RL values can give skewed FL/RL ratios. **List of genes highlighted in red circle in Figure 3D have high RL values, many of which have unaffected FL values. All values are robust Z-scores averaged over three replicates. Click here for file

Additional file 4 **Hits identified in the SRSF screen are also present in the HFA collection. **List of hits identified in SRSF screen, as shown in Table 2, but including dsRNA amplicon names for SRSF and HFA libraries, as well as Flybase IDs. Click here for file

Additional file 5 **Tertiary screening results and primer design. **List of the hits identified in both SRSF and HFA screens. Included are Z-scores calculated from FL/RL ratios and averaged across triplicates, for genome and tertiary screens. Significance >2 or <−2 highlighted in grey and bold, significance >1.7 or <-1.7 highlighted in blue and mark #. All sequence information is included where novel designs were made. Some genes were not screened (NS) in the tertiary screen either due to inability to target independent regions, independent regions had already been screened, or the gene is now withdrawn from Flybase. Click here for file

Additional file 6 **The 6 genes found to be significant in both SRSF and Baeg screens. **Fold change values are shown as originally presented in ref [15], and +/- indicates an increase or decrease in reporter activity, respectively. Grey/blue boxes highlight significance levels as indicated in key. Click here for file
