# Supplementary material for: Advances in genome-wide RNAi cellular screens: a case study using the Drosophila JAK/STAT pathway
Source: BMC Genomics. 2012 Sep 24;13:506. doi: 10.1186/1471-2164-13-506 (PMC3526451; doi:10.1186/1471-2164-13-506)
Supplement: Additional file 1 — Plate layouts of HFA and SRSFv1 libraries. (A) Layout of HFA library plates including the position of positive (blue) and negative pathway regulators, used as controls in the Müller et al. screen. (B) Layout of SRSFv1 library including the DIAP1 barcode (black), technical controls (purple), non-interacting controls (yellow) and positive (blue) and negative (red) pathway regulators used as controls. [file 1471-2164-13-506-S1.pdf]

Additional File 1

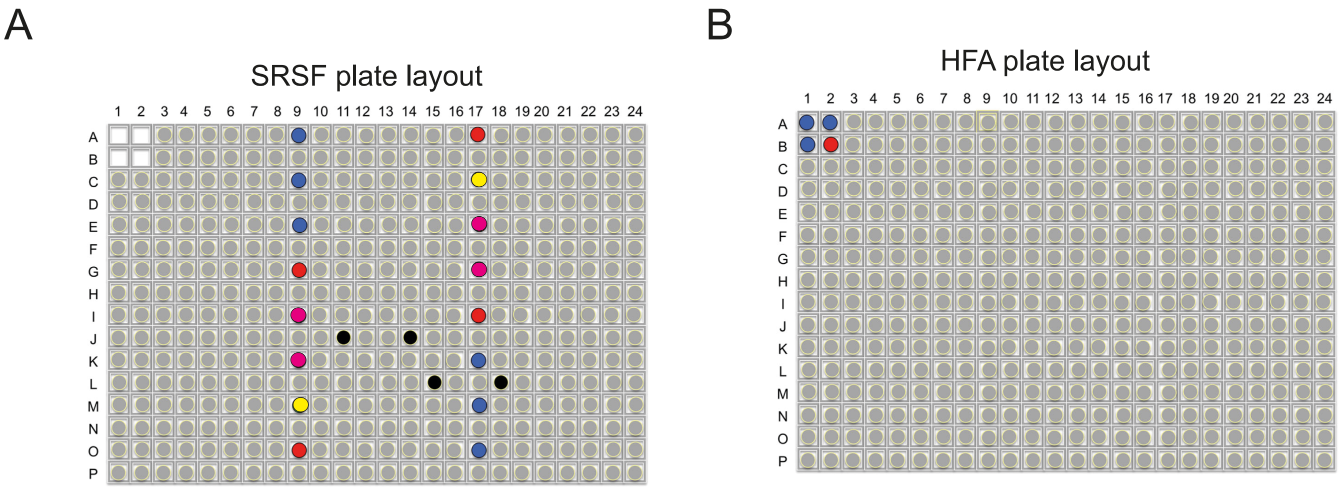

KEY:
- dsRNA
- barcode (e.g. DIAP1)
- non-interacting controls (e.g. ZK686.3)
- technical control (e.g. RLuc)
- positive pathway regulator (e.g. Dome)
- negative pathway regulator (e.g. Socs36E)
