# Supplementary figures and images for: Advances in genome-wide RNAi cellular screens: a case study using the Drosophila JAK/STAT pathway
Source: BMC Genomics. 2012 Sep 24;13:506. doi: 10.1186/1471-2164-13-506 (PMC3526451; doi:10.1186/1471-2164-13-506)

# Additional File 2

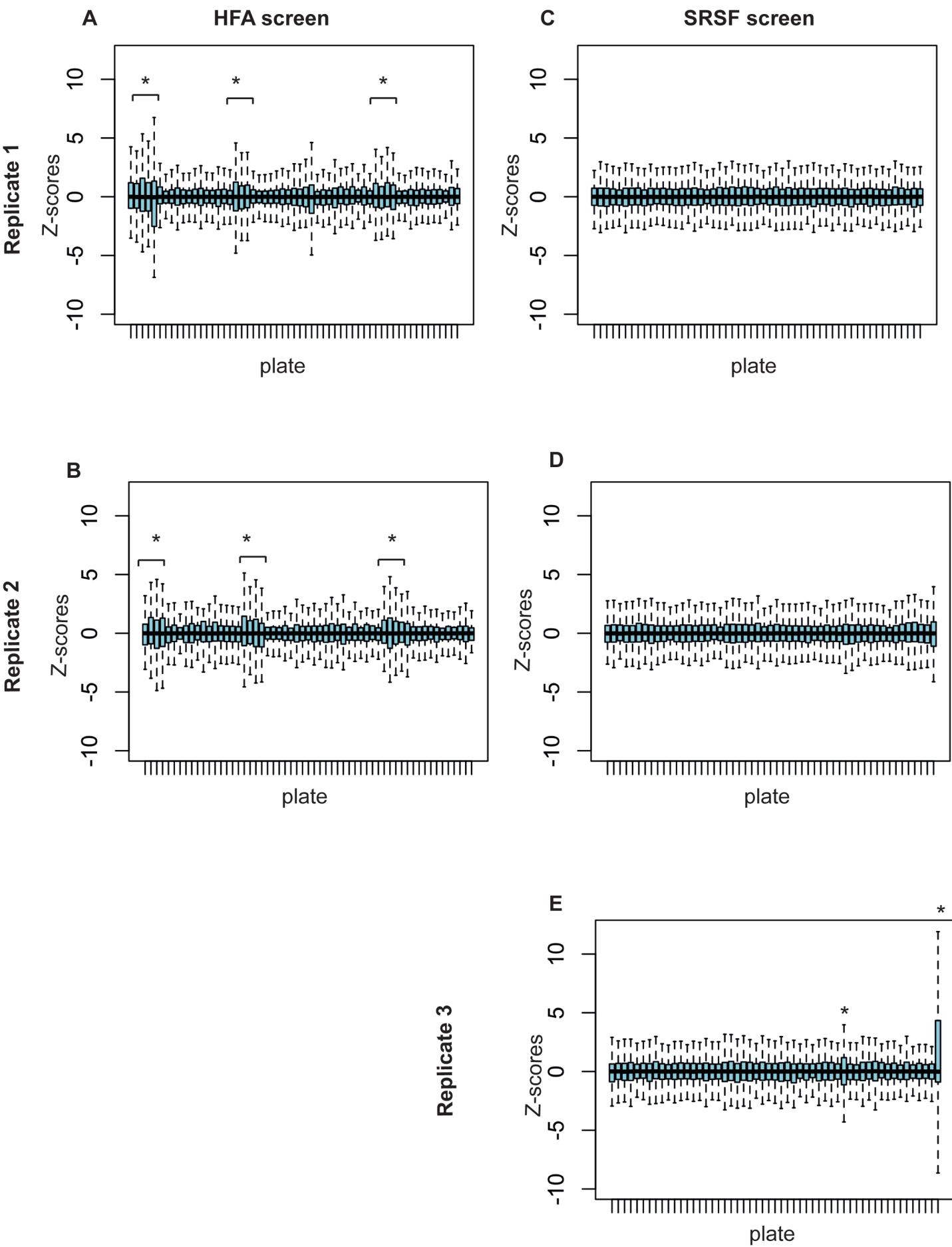

Supplement: Additional file 2 — Quality control of HFA and SRSF JAK/STAT screens. Box and whisker plots representing each plate from separate replicates in HFA and SRSF screens. Asterisks denote plates where variance can be observed by eye. [file 1471-2164-13-506-S2.pdf]
