# Supplementary material for: Advances in genome-wide RNAi cellular screens: a case study using the Drosophila JAK/STAT pathway
Source: BMC Genomics. 2012 Sep 24;13:506. doi: 10.1186/1471-2164-13-506 (PMC3526451; doi:10.1186/1471-2164-13-506)
Supplement: Additional file 3 — dsRNAs resulting in high RL values can give skewed FL/RL ratios. List of genes highlighted in red circle in Figure 3D have high RL values, many of which have unaffected FL values. All values are robust Z-scores averaged over three replicates. [file 1471-2164-13-506-S3.pdf]

Additional File 3

SYMBOL	GO_BIOLOGICAL_PROCESS	BKN	FL_Zscore	RL_Zscore	FL/RL_Zscore
Hsp70Bb	heat shock-mediated polytene chromosome puffing ; response	BKN40659	-1.7	25.6	-14.7
Hsp70Aa	heat shock-mediated polytene chromosome puffing ; response	BKN40468	-1.2	25.5	-14.1
Hsp70Bbb	heat shock-mediated polytene chromosome puffing ; response	BKN42219	-1.3	23.5	-13.3
Hsp70Bc	heat shock-mediated polytene chromosome puffing ; response	BKN41955	-0.6	20.9	-11.3
Hsc70-2	protein folding ; response to heat ; protein folding	BKN45569	-1.0	18.6	-10.4
Tim17b1	protein transport ; protein targeting to mitochondrion	BKN32748	0.4	17.1	-8.2
CG5800	larval somatic muscle development ; neurogenesis	BKN22766	0.0	16.1	-8.1
Hsc70-2	protein folding ; response to heat ; protein folding	BKN29772	-1.2	15.7	-9.0
Dhpr	metabolic process	BKN28477	-0.3	14.8	-7.9
yellow-g	biological_process	BKN29964	-1.3	14.7	-8.7
CG3328	regulation of transcription, DNA-dependent	BKN27762	0.1	14.5	-7.1
CG11370	-	BKN29963	-0.2	14.2	-7.4
Hsc70-1	protein folding ; response to heat ; protein folding	BKN31450	-0.8	14.1	-7.7
Or59b	sensory perception of smell ; sensory perception of smell	BKN30129	-0.5	14.0	-7.6
Hsp83	spermatogenesis ; centrosome cycle ; protein folding ; R7 cell	BKN21307	-1.5	13.6	-8.4
Prosalpha5	ubiquitin-dependent protein catabolic process ; cellular process	BKN28214	0.5	13.0	-6.3
sec10	neurotransmitter secretion ; synaptic vesicle docking involved i	BKN20533	-0.2	12.4	-6.4
mats	signal transduction ; cell proliferation ; apoptotic process	BKN20236	0.5	12.4	-5.6
Mtr3	regulation of gene expression	BKN45761	-0.8	11.9	-6.8
CG16890	-	BKN28865	0.9	11.0	-4.8
CG30156	neurogenesis ; protein folding	BKN45189	-1.0	11.0	-6.5
Pros26	ubiquitin-dependent protein catabolic process ; proteolysis ; pr	BKN22072	-0.3	10.5	-5.6
Hsp70Ba	heat shock-mediated polytene chromosome puffing ; response	BKN41583	0.3	10.3	-4.9
Prosbeta5	centrosome organization ; mitotic spindle organization ; mitotic	BKN45135	0.1	10.2	-5.2
Dscam4	cell adhesion	BKN42505	0.5	9.0	-3.9
CG34423	negative regulation of nucleotide metabolic process	BKN40085	0.6	8.8	-3.7
CG7384	-	BKN24987	0.6	8.8	-3.8
CG9130	-	BKN40033	0.4	8.7	-3.5
DnaJ-1	response to heat ; response to heat ;protein folding	BKN45366	-1.1	8.6	-5.4
Obp49a	sensory perception of chemical stimulus	BKN25372	-0.5	8.4	-4.7
IntS6	-	BKN45482	-0.6	8.4	-4.8
Rep	intracellular protein transport ; neurotransmitter secretion ; ve	BKN21378	-0.6	8.1	-4.7
Prosbeta3	proteolysis ; response to DNA damage stimulus ; neurogenesis	BKN28041	0.1	7.9	-3.9
Rpt1	proteolysis ; cellular process ; response to DNA damage stimul	BKN20388	0.5	7.7	-3.6
Nuf2	mitotic metaphase plate congression ; chromosome segregatio	BKN27199	0.2	7.4	-3.5
tRNA:CR31130	translation	BKN40453	1.4	7.4	-1.6
Prosbeta5	centrosome organization ; mitotic spindle organization ; mitotic	BKN21455	-0.3	7.4	-4.1
CG3906	-	BKN25623	-0.4	7.3	-4.0
CG32425	-	BKN40467	0.3	7.2	-3.4
CG14870	cilium assembly	BKN29337	0.1	7.2	-3.4
Fas3	axon guidanc; synaptic target recognition ; ovarian follicle cell	BKN23309	-0.2	7.1	-3.6
DnaJ-1	response to heat ; response to heat ; protein folding	BKN31167	0.1	6.9	-3.2
Hsc70Cb	protein folding	BKN20891	0.6	6.7	-2.6
RpL13A	translation ; translation ; Notch signaling pathway ; chaeta mo	BKN40429	0.2	6.7	-2.4
CG8543	-	BKN33113	1.9	6.7	-0.8
CG13297	-	BKN33121	1.1	6.6	-1.5
Obp47a	sensory perception of chemical stimulus ; sensory perception o	BKN31646	0.7	6.6	-2.5
dro2	defense response ; defense response to fungus	BKN42373	1.3	6.6	-1.2
CG8547	-	BKN46009	0.3	6.5	-2.8
mamo	female meiosis ; sperm chromatin decondensation ; gamete ge	BKN33149	1.3	6.4	-1.4
mit(1)15	mitotic sister chromatid segregation ; meiotic anaphase I ; mit	BKN20532	0.6	6.4	-2.6
snoRNA:Me28S-(	-	BKN40401	1.1	6.3	-1.3
snRNA:U1:95Cb	nuclear mRNA splicing, via spliceosome	BKN40449	1.9	6.2	-0.5
CG33494	-	BKN33101	1.3	6.2	-1.0
Brd8	negative regulation of gene expression	BKN27830	1.3	6.2	-1.8
CG17202	signal transduction	BKN29787	0.1	6.1	-2.9
CG15478	-	BKN33133	0.2	6.1	-2.2
Rpn1	proteolysis ; mitotic spindle organization ; mitotic spindle elong	BKN22968	-0.5	6.1	-3.6
snRNA:U5:23D	nuclear mRNA splicing, via spliceosome	BKN42345	1.3	6.0	-0.9
CG15649	-	BKN33097	0.7	6.0	-1.5
Prosbeta7	cell proliferation ; mitotic spindle elongation ; mitotic spindle o	BKN28365	-0.6	5.9	-3.6
Pros26.4	proteolysis ; ubiquitin-dependent protein catabolic process ;cel	BKN21532	-0.4	5.8	-3.5
CG6154	proteolysis	BKN27428	-0.5	5.8	-3.4
CG40040	-	BKN40045	0.6	5.7	-1.5
TwdlU	chitin-based cuticle development ; body morphogenesis	BKN30817	1.2	5.7	-1.0
san	mitotic sister chromatid cohesion ; mitosis ; lateral inhibition	BKN46596	0.7	5.7	-2.4
CG18823	-	BKN42325	1.3	5.7	-0.8
CG41427	-	BKN42705	1.5	5.7	-0.7
CG9517	oxidation-reduction process ; alcohol metabolic process	BKN30833	0.9	5.6	-1.3
gfzf	neurogenesis ; mitotic cell cycle G2/M transition DNA damage c	BKN33145	0.2	5.6	-2.1
CG33307	biological_proces	BKN30797	0.8	5.6	-1.3
CR41583	-	BKN42729	1.5	5.6	-0.6
betaTub85D	microtubule-based process ; salivary gland morphogenesis ; m	BKN30793	1.9	5.6	-0.2
lmd	regulation of transcription, DNA-dependent ; somatic muscle de	BKN27849	0.7	5.5	-2.0
CG30283	proteolysis	BKN33073	0.9	5.5	-1.1
And	detection of calcium ion	BKN32753	1.5	5.3	-0.5
tRNA:L:35C	translation	BKN42765	1.9	5.3	-0.2
CG11866	-	BKN33109	1.3	5.3	-0.7
Hsp68	determination of adult lifespan ; response to heat ; response to	BKN25780	-0.2	5.3	-2.8
Cpr67Fb	-	BKN29517	0.8	5.3	-1.7
CG34193	-	BKN42361	1.6	5.3	-0.3
CG7039	small GTPase mediated signal transduction	BKN28403	-0.6	5.1	-3.3
Tbp-1	proteolysis ; cellular process ; protein catabolic process	BKN21879	0.1	5.1	-2.6
Rpt4	proteolysis ; neurogenesis ; protein catabolic process	BKN29219	-0.5	5.0	-3.1
CG7377	-	BKN42709	1.5	5.0	-0.2

= Z-score <-2

= Z-score >2
