# Supplementary material for: Advances in genome-wide RNAi cellular screens: a case study using the Drosophila JAK/STAT pathway
Source: BMC Genomics. 2012 Sep 24;13:506. doi: 10.1186/1471-2164-13-506 (PMC3526451; doi:10.1186/1471-2164-13-506)
Supplement: Additional file 4 — Hits identified in the SRSF screen are also present in the HFA collection. List of hits identified in SRSF screen, as shown in Table 2, but including dsRNA amplicon names for SRSF and HFA libraries, as well as Flybase IDs. [file 1471-2164-13-506-S4.pdf]

Additional File 4

Gene	BKN	HFA	Flybase ID
dome	BKN25660	HFA19583	FBgn0043903
Stat92E	BKN20615	HFA16870	FBgn0016917
hop	BKN24272	HFA20340	FBgn0004864
Tbp	BKN20836	HFA04662	FBgn0003687
mask	BKN20625	HFA15370	FBgn0043884
TfIIA-L	BKN28669	HFA16882	FBgn0011289
CG40121	BKN41059	HFA18531	FBgn0058121
dom	BKN21379	HFA04558	FBgn0020306
Mov34	BKN21370	HFA04624	FBgn0002787
E2f	BKN20400	HFA16655	FBgn0011766
CG32269	BKN40155	HFA08195	FBgn0052269
RpL24	BKN28312	HFA03185	FBgn0032518
ctrip	BKN20611	HFA12242	FBgn0260794
RpLP2	BKN27368	HFA07539	FBgn0003274
Chd1	BKN21926	HFA00745	FBgn0250786
Dp	BKN29434	HFA07402	FBgn0011763
RpII215	BKN20898	HFA20280	FBgn0003277
Dcp2	BKN21841	HFA10597	FBgn0036534
CG9723	BKN21228	HFA20206	FBgn0030768
pncr017:3R	BKN31481	HFA14080	FBgn0046756
Cnot4	BKN22063	HFA02830	FBgn0051716
CG11873	BKN26700	HFA00063	FBgn0039633
qkr54B	BKN27961	HFA07670	FBgn0022987
kis	BKN20986	HFA00823	FBgn0086902
shrb	BKN28557	HFA07061	FBgn0086656
ftz-f1	BKN28995	HFA12996	FBgn0001078
TSG101	BKN28961	HFA11098	FBgn0036666
Ptp61F	BKN21934	HFA08683	FBgn0003138
Saf-B	BKN20574	HFA16167	FBgn0039229
l(3)mbt	BKN20908	HFA16983	FBgn0002441
CG7185	BKN28487	HFA10781	FBgn0035872
Sin	BKN22002	HFA11880	FBgn0028402
Hsp60B	BKN30688	HFA00542	FBgn0011244
CG11399	BKN21417	HFA19621	FBgn0037021
Socs36E	BKN45944	HFA02455	FBgn0041184
lola	BKN30256	HFA06741	FBgn0005630
Surf4	BKN22052	HFA16875	FBgn0019925
CkIalpha	BKN27574	HFA20231	FBgn0015024
ham	BKN22290	HFA02070	FBgn0045852
zfh1	BKN29931	HFA17098	FBgn0004606
chinmo	BKN45751	HFA00509	FBgn0086758
srp	BKN45799	HFA18886	FBgn0003507
