# Supplementary material for: Advances in genome-wide RNAi cellular screens: a case study using the Drosophila JAK/STAT pathway
Source: BMC Genomics. 2012 Sep 24;13:506. doi: 10.1186/1471-2164-13-506 (PMC3526451; doi:10.1186/1471-2164-13-506)
Supplement: Additional file 6 — The 6 genes found to be significant in both SRSF and Baeg screens. Fold change values are shown as originally presented in ref [15], and +/- indicates an increase or decrease in reporter activity, respectively. Grey/blue boxes highlight significance levels as indicated in key. [file 1471-2164-13-506-S6.pdf]

Additional File 6

Row	Gene	SRSF genome	SRSF secondary	Baeg genome (direction and fold change)
1	*dome*	**-7.9**	**-7.1**	**- >10**
2	*Stat92E*	**-6.8**	**-11.4**	**- >10**
3	*hop*	**-5.7**	**-10**	**- >10**
7	*Ptp61F*	**2.3**	**3.6**	**+ >2.5**
13	*Socs36E*	**2.8**	1.8 #	**+ >3**
26	*lola*	**3.1**	**4**	**+ >3**

Key:

grey and bold = Z-score <-2 or >2

blue and # = Z-score <-1.7 or >1.7
